# Editorial: Psychological mechanisms and mental health in the face of current conflicts and crises

**DOI:** 10.3389/fpubh.2025.1684360

**Published:** 2025-09-04

**Authors:** Hubert Annen

**Affiliations:** Military Academy, ETH Zurich, Birmensdorf, Switzerland

**Keywords:** crisis, crisis management, intervention, resilience, support mechanisms

## Introduction

Recent years have been marked by a confluence of global crises, such as pandemics, war, and ecological threats, that have reshaped psychological landscapes for individuals and communities worldwide. These multifaceted challenges have not only threatened public health and safety but have also profoundly tested emotional resilience, strained moral values, and revealed systemic vulnerabilities in mental health support. The present Research Topic, “*Psychological Responses to Conflict and Crisis,”* brings together a diverse set of investigations that collectively explore how people experience, respond to, and adapt to extraordinary adversity.

This collection of 11 articles spans a rich array of contexts and underscores the importance of multidisciplinary approaches to crisis psychology. Together, these contributions provide fresh insights into protective mechanisms, psychosocial risk factors, and the complex interplay between individual agency and institutional environments.

## Crisis contexts: refugee experience, conflict zones, and global anxiety

Several studies examine how geopolitical upheaval and global threats affect mental health, especially in populations indirectly or directly exposed to conflict. Kovács et al. explore the psychological outcomes of Ukrainian refugees in Hungary and Poland. Their findings highlight the buffering role of social support in shaping attitudes and acculturation among displaced individuals, even when trauma exposure is high. Similarly, Prazeres et al. investigate anxiety in the Portuguese population, comparing fears related to COVID-19 and nuclear war. Their work illustrates how distant crises can nonetheless provoke significant psychological distress in non-combat populations.

Stieger et al. offer a rare, *in situ* analysis of war exposure in Ukraine using an experience-sampling approach. They show that civilians frequently exposed to sirens and explosions continue to experience heightened anxiety and somatic distress, thus confirming the persistent toll of existential threat and the limited protective effects of habituation.

Adding to the understanding of individual responses to war, Wójtowicz-Szefler et al. explore resilience and value changes in Polish volunteers assisting Ukrainian refugees. Their work uncovers that volunteering during the war reinforces personal values and fosters resilience, highlighting a reciprocal relationship between prosocial action and psychological adaptation.

## Mental health in uniformed services: moral injury, combat exposure, and compassion fatigue

Military contexts are prominently represented in this Research Topic. Wesemann et al. conduct a quasi-experimental study on German soldiers deployed to Afghanistan, finding significantly elevated rates of PTSD, anxiety, and depression among those exposed to life-threatening incidents. These findings stress the need to distinguish between general deployment stress and the acute psychological consequences of traumatic threat.

Levy and Gross contribute a qualitative exploration of Israeli veterans' post-combat identity reconstruction. They distinguish between two narratives, namely humanitarian and national security, that shape the trajectory of moral injury and civic engagement. Veterans expressing humanitarian guilt often seek amends through peace advocacy, while those embedded in a national security frame may engage in activism from a critical stance. This study broadens our understanding of moral injury beyond clinical symptomatology, illuminating its transformative social potential.

Valladares-Garrido et al. explore fear of COVID-19 among Peruvian military personnel, revealing that younger age, sleep deprivation, and pre-existing mental health challenges predict heightened distress. These findings signal the compounded psychological risk faced by military personnel tasked with public health duties during crises.

## Healthcare workers and the frontlines of emotional burden

The COVID-19 pandemic has placed extraordinary pressure on healthcare systems, particularly frontline personnel. Zhang et al. examine the role of role stress in generating compassion fatigue among nurses in China and identify perceived social support and emotion regulation efficacy as key mediators. With 94.2% of nurses experiencing some level of compassion fatigue, this study highlights the urgent need for systemic support structures and targeted interventions.

Neil-Sztramko et al. provide a systematic review of mental health interventions for frontline healthcare workers during public health emergencies. Their findings point to the effectiveness of interventions such as psychotherapy, psychoeducation, and mind-body practices, underscoring the necessity of proactive institutional planning in crisis preparedness.

## Civilians under stress: preparedness, perception, and resilience

Outside direct combat and healthcare contexts, two contributions emphasize civilian responses to crises. Faryabi et al. apply Protection Motivation Theory to examine disaster preparedness in Iranian households. Their study identifies fear and perceived vulnerability as strong motivators for adopting protective behaviors, providing actionable insight for public risk communication strategies.

Massag et al. trace psychological distress in Germany during the Ukraine war, revealing that although overall distress levels declined over time, a notable proportion of respondents continued to experience clinically significant symptoms. The study illustrates the ripple effects of distant wars on populations psychologically proximate through media exposure and geopolitical concerns.

## Synthesizing insights: moral, emotional, and structural dimensions

Several cross-cutting themes emerge across this volume. First, the significance of moral reasoning and identity in shaping crisis responses, particularly in contexts involving conflict, caregiving, and trauma, underscores the need for frameworks that integrate ethical, psychological, and sociocultural dimensions of resilience.

Second, the recurring role of social support and emotional regulation across diverse populations, from nurses and veterans to civilians and volunteers, reinforces the universality of these protective factors. Whether explicitly measured (as in Zhang et al. and Kovács et al.) or implicitly noted (as in Wójtowicz-Szefler et al.), these elements are crucial levers for psychological interventions.

Finally, the studies collectively emphasize that crisis-related distress is neither evenly distributed nor inevitable. Institutional, social, and personal resources play mediating roles in shaping individual outcomes. This insight shifts the focus from passive coping to active, policy-informed engagement with mental health support, especially among at-risk populations like refugees, healthcare workers, and soldiers.

## Conclusion

The articles in this Research Topic collectively advance our understanding of how individuals and groups navigate crises, for instance, through suffering, adaptation, meaning-making, and, in many cases, growth. As global uncertainties persist, these insights offer not only diagnostic clarity but also hope: that resilience can be cultivated, that moral strain can lead to action, and that even in the darkest moments of collective experience, psychological science can offer paths to recovery, connection, and renewed purpose. The work represented here reminds us that while crises expose the limits of our systems and the fragility of our lives, they also illuminate the extraordinary human capacity to adapt and to care. Moving forward, it will be critical for researchers, policymakers, and practitioners to translate these findings into tangible frameworks for prevention, early intervention, and long-term support. Only by combining scientific understanding with collective responsibility can we ensure that the psychological consequences of conflict and crisis do not define us—but rather, that our responses to them shape a more resilient, compassionate future.

